# Prognostic significance of HALP (hemoglobin, albumin, lymphocyte and platelet) in patients with bladder cancer after radical cystectomy

**DOI:** 10.1038/s41598-018-19146-y

**Published:** 2018-01-15

**Authors:** Ding Peng, Cui-jian Zhang, Yan-qing Gong, Han Hao, Bao Guan, Xue-song Li, Li-qun Zhou

**Affiliations:** 1 Department of Urology, Peking University First Hospital, Beijing, China; 2 Institute of Urology, Peking University, Beijing, China; 3 National Urological Cancer Center, Beijing, China; 4 Urogenital Diseases (male) Molecular Diagnosis and Treatment Center, Peking University, Beijing, China

## Abstract

The outcome of bladder cancer after radical cystectomy is heterogeneous. We aim to evaluate the prognostic value of HALP (hemoglobin, albumin, lymphocyte and platelet) and explore novel prognostic indexes for patients with bladder cancer after radical cystectomy. In this retrospective study, 516 patients with bladder cancer after radical cystectomy were included. The median follow-up was 37 months (2 to 99 mo). Risk factors of decreased overall survival were older age, high TNM stage, high American Society of Anesthesiologists (ASA) grade and low HALP score. The predictive accuracy was better with HALP-based nomogram than TNM stage (C- index 0.76 ± 0.039 vs. 0.708 ± 0.041). By combining ASA grade and HALP, we created a novel index—HALPA score and found it an independent risk factor for decreased survival (HALPA score = 1, HR 1.624, 95% CI 1.139–2.314, P = 0.007; HALPA score = 2, HR 3.471, 95% CI: 1.861–6.472, P < 0.001).The present study identified the prognostic value of HALP and provided a novel index HALPA score for bladder cancer after radical cystectomy.

## Introduction

Bladder cancer is the most common cancer in the urogenital system^1^. In 2017, there were 79030 new cases estimated in United States; the cancer ranks 6th in all cancers and 4th in males^2^. Radical cystectomy is the typical treatment for bladder cancer including muscle-invasive bladder cancer (MIBC) and high risk non-MIBC (NMIBC)^1,3,4^. Despite the advancements in surgical techniques and chemotherapy, the outcome of bladder cancer is poor, especially for patients with advanced and metastatic disease^3,5^.

The outcome of bladder cancer is heterogeneous, with 77.9% survival at 5 years for patients at all stages, 96.4% with *in situ* disease, 70.2% with invasive tumors and 3.0% and 5.4% with regional and distant-stage disease^6^. Therefore, it is crucial to stratify the risk of mortality and establish the optimal therapy and follow-up strategies.

Besides the traditional TNM system, numerous prognostic factors may predict outcome with bladder cancer. Haematological parameters including leukocyte count (neutrophil, lymphocyte and monocyte), platelet count and levels of hemoglobin, albumin, C-reactive protein (CRP), and fibrinogen are all easily assessed and reliable indicators of postoperative prognosis^7–11^. The combination of those indices have better predictive ability, for example, neutrophil to lymphocyte ratio (NLR), platelet to lymphocyte ratio (PLR) and lymphocyte to monocyte ratio (LMR)^12–14^. Recently, a novel index, HALP (hemoglobin, albumin, lymphocyte and platelet levels) was developed and demonstrated as a significant prognostic factor for patients with colorectal cancer and gastric carcinoma^15,16^.

Here we evaluated the prognostic value of HALP and explored the development of a novel prognostic index for patients with bladder cancer after radical cystectomy.

## Results

### Patient characteristics

A total of 516 patients were enrolled in this study (436 84.5% males; median age 66 years) (Table 1). The median follow-up was 37 months (interquartile range [IQR] 20–56). 91 patients (17.6%) received adjuvant chemotherapy after radical cystectomy. At the end of follow-up, 164 patients (31.8%) had died from any cause and the 3- and 5-year estimated overall survival was 75.3% and 69%. Hypoalbuminemia was present in 8.3% patients (n = 43; 3.9% with NMIBC, 11.1% with MIBC, P = 0.009) and anemia was present in 27.9% (n = 144; 12.4% with NMIBC, 37.5% with MIBC, P < 0.001). Other clinicopathological characteristics are in Table 1.

**Table 1:** Clinicopathological characteristics for 516 bladder cancer patients.

Characteristics	
Age, median (IQR)	66 (57–73)
Gender	
female	80 (15.5%)
male	436 (84.5%)
Smoking history	
no	355 (68.8%)
yes	161 (31.2%)
Alcohol-drinking history	
no	449 (87.0%)
yes	67 (13%)
Hypertension	
no	367 (71.1%)
yes	149 (28.9%)
Histology subtype	
transitional cell carcinoma	488 (94.6%)
non-transitional cell carcinoma	28 (5.4%)
Grade	
2	126 (24.4%)
3	385 (75.6%)
T-stage	
NMIBC	162 (31.4%)
MIBC	354 (68.6%)
N-stage	
negative	81 (15.7%)
positive	435 (84.3%)
M-stage	
negative	511 (99.03%)
positive	5 (0.97%)
Adjunctive chemotherapy	
yes	91 (17.6%)
no	425 (82.4%)
ASA grade	
1&2	436 (84.5%)
3&4	80 (15.5%)
Anemia	
present	144 (27.9%)
absent	342 (72.1%)
Hypoalbuminemia	
present	43 (8.3%)
absent	442 (85.7%)
PLR, median (IQR)	133.8 (98.22–180.23)
HALP, median (IQR)	41.2 (27.78–58.71)

IQR, interquartile range; MIBC, muscle-invasive bladder cancer; NMIBC, non-muscle invasive bladder cancer; ASA, American Society of Anesthesiologists; PLR: platelet to lymphocyte ratio.

### Association between clinicopathological features and HALP

**Figure 1:**
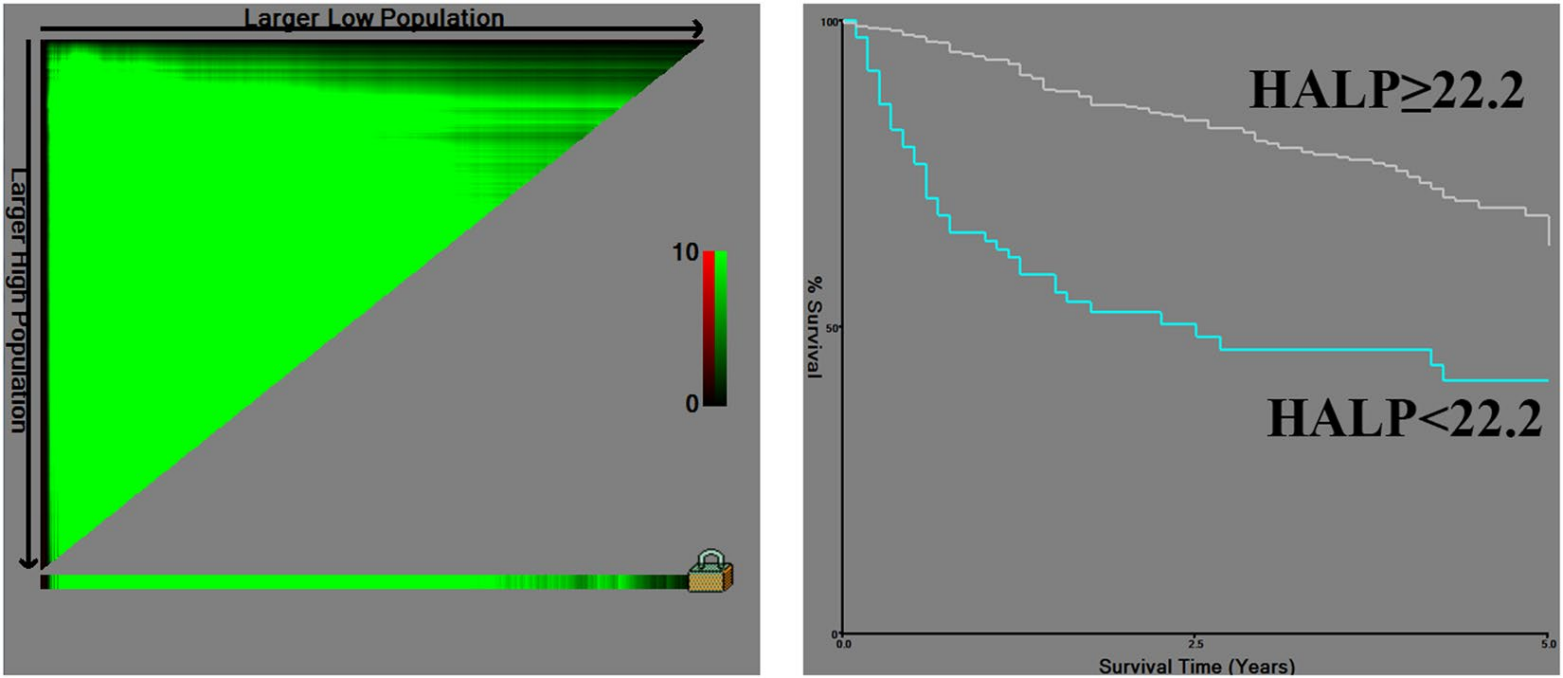
Cut off value for HALP by X-tile software.

We determined the cut-off values of HALP as 22.2 and PLR as 214.8. Patients were divided into 2 groups according to the cut-offs (Fig. 1). Older age, female sex, high T stage, high ASA grade and anemia were associated with low HALP score (Table 2).

**Table 2:** Association between clinicopathological characteristics and HALP.

Cohort characteristics	HALP value		
	Low	High	P value
Age			**0.019**
<65	24(32.9)	196(47.7)	
≥65	49(67.1)	215(52.3)	
Gender			**0.013**
female	18(24.7)	55(13.4)	
male	55(75.3)	356(86.6)	
Smoking history			0.204
no	55(75.3)	279(67.9)	
yes	18(24.7)	132(32.1)	
Alcohol-drinking history			0.646
no	65(89.0)	358(87.1)	
yes	8(11.0)	53(12.9)	
Hypertension			0.890
no	52(71.2)	296(72.0)	
yes	21(28.8)	115(28.0)	
Histology type			0.553
transitional cell carcinoma	70(95.9)	387(94.2)	
non-transitional cell carcinoma	3(4.1)	24(5.8)	
Grade			0.178
2	13(17.8)	102(24.8)	
3	60(82.2)	304(74.0)	
T-stage			**<0.001**
NMIBC	7(9.6)	146(35.5)	
MIBC	66(90.4)	265(64.5)	
N-stage			0.725
negative	61(83.6)	350(85.2)	
positive	12(16.4)	61(14.8)	
M-stage			0.026
negative	70(95.9)	409(99.5)	
positive	3(4.1)	2(0.5)	
Adjunctive chemotherapy			0.170
yes	16(21.9)	68(16.5)	
no	57(78.1)	343(83.5)	
ASA grade			**0.010**
1&2	54(74.0)	353(85.9)	
3&4	19(26.0)	58(14.1)	
Anemia			**<0.001**
absent	13(17.8)	327(79.6)	
present	60(82.2)	84(20.4)	
Hypoalbuminemia			**<0.001**
absent	47(64.4)	394(95.9)	
present	26(35.6)	17(4.1)	
PLR			**<0.001**
low	23(31.5)	397(96.6)	
high	50(68.5)	14(3.4)	

Data are shown as n (%).

MIBC, muscle-invasive bladder cancer; NMIBC, non-muscle invasive bladder cancer; ASA, American Society of Anesthesiologists; PLR: platelet to lymphocyte ratio.

### Prognostic value of HALP for outcomes

**Figure 2:**
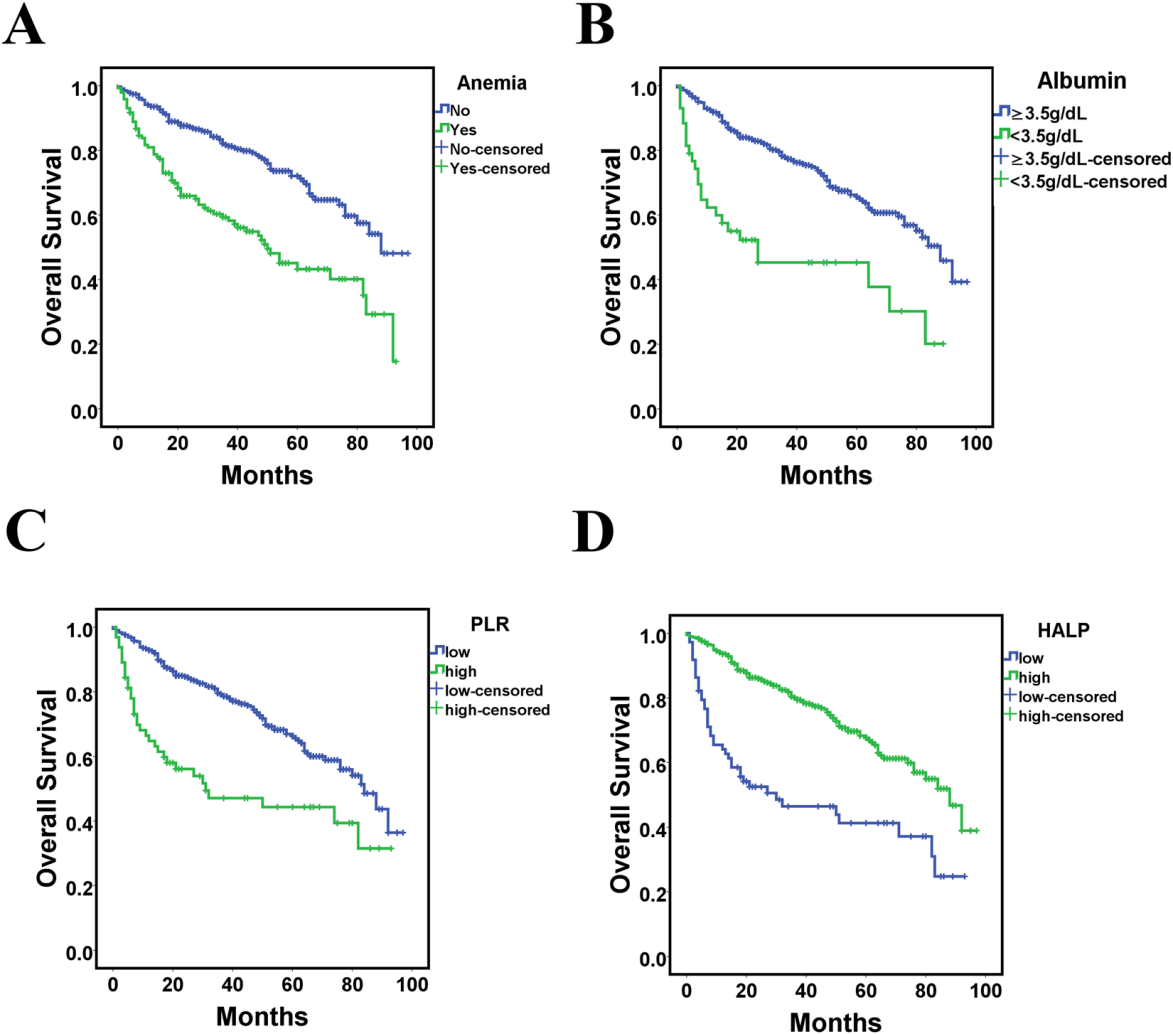
Kaplan-Meier curves for overall survival in patients with bladder cancer according to anemia level, (**A**) hypoalbuminemia level, (**B**) platelet to lymphocyte rate (PLR), (**C**) and HALP (**D**).

On univariate analysis, hypoalbuminemia, anemia, high PLR, and low HALP were all associated with worse overall survial (Fig. 2).Other factors included older age (>65 years), high TNM grade and high ASA grade (all P < 0.05) (Table 3). These variables were included in a multivariable analysis. Independent factors associated with decreased overall survival were older age, high TNM stage, high ASA grade and low HALP score (HR = 1.986, 95% CI: 1.386–2.886, P < 0.001) for bladder cancer patients after radical cystectomy (Table 3).

**Table 3:** Univariate and multivariable analysis of factors associated with overall survival in bladder cancer patients who underwent radical cystectomy.

Variable	Univariate analysis		Multivariate analysis	
	HR (95% CI)	*P* value	HR (95% CI)	*P* value
Gender (female vs. male)	0.794(0.528–1.193)	0.264		
Age (>65 vs. ≤65)	2.220(1.592–3.098)	**0.001**	1.923 (1.336–2.768)	**<0.001**
Smoking history	1.014(0.725–1.417)	0.937		
Alcohol-drinking history	0.922(0.565–1.504)	0.743		
Hypertension	1.130(0.803–1.591)	0.480		
Histology type (TCC vs. no-TCC)	0.706(0.331)1.506	0.363		
G (3 vs. 2)	2.118(1.373–3.267)	**<0.001**		
T (MIBC vs. NMIBC)	4.301(2.665–6.942)	**<0.001**	2.769 (1.679–4.565)	**<0.001**
N (positive vs. negative)	2.448(1.716–3.491)	**<0.001**	2.114 (1.434–3.116)	**<0.001**
M (positive vs. negative)	10.509(3.842–28.746)	**<0.001**	4.153 (1.454–11.863)	**0.008**
Adjunctive chemotherapy	2.056(1.437–2.941)	**<0.001**		
ASA (3&4 vs. 1&2)	2.068(1.442–2.964)	**<0.001**	1.540(1.038–2.284	**0.032**
Hypoalbuminemia	2.919(1.900–4.484)	**<0.001**		
Anemia	2.433(1.775–3.335)	**<0.001**		
PLR	1.963(1.421–2.712)	**<0.001**		
HALP	2.820(1.976–4.025)	**<0.001**	1.986 (1.386–2.886)	**<0.001**

TCC, transitional cell carcinoma; MIBC, muscle-invasive bladder cancer; NMIBC, non-muscle invasive bladder cancer; ASA, American Society of Anesthesiologists; PLR: platelet-lymphocyte ratio; HR, hazard ratio; CI: confidence interval.

### HALP-based risk model for bladder cancer patients after radical cystectomy

**Figure 3:**
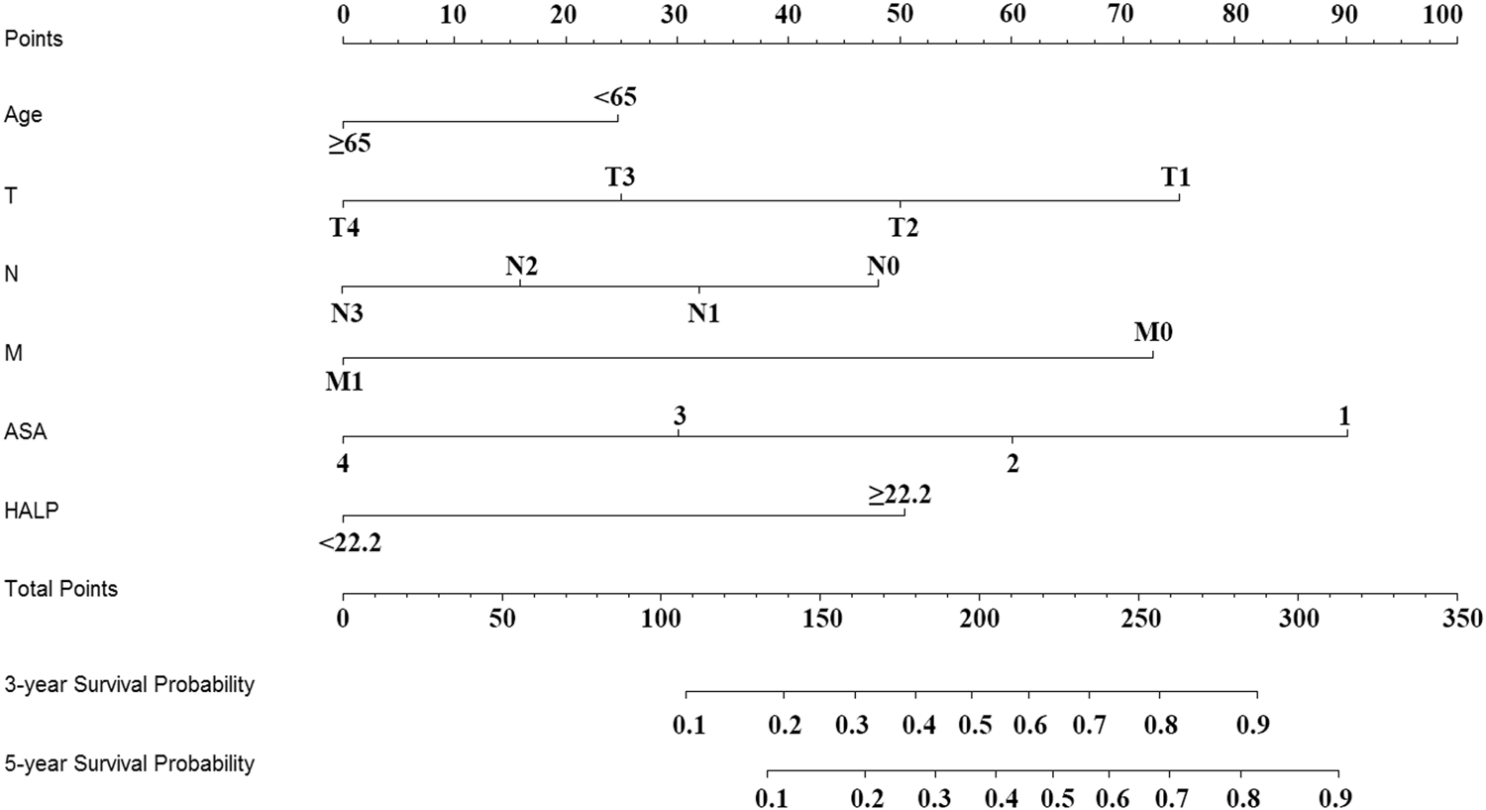
Nomogram for 3-year and 5-year survival.

**Figure 4:**
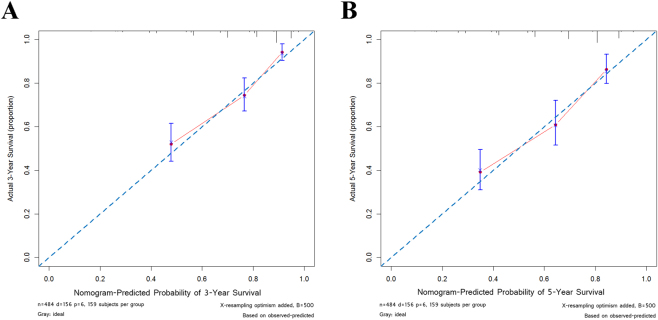
Calibration curves for 3-year (**A**) and 5-year (**B**) survival.

To further assess the prognostic ability of HALP and other variables, we used nomogram including the independent risk factors in the multivariable regression analysis (Fig. 3). Predicted 3- and 5- year survival was similar to the actual rates (Fig. 4). The predictive accuracy of C-indices was 0.76 ± 0.039 for the HALP-based nomogram as compared with 0.708 ± 0.041 for the TNM stage-based nomogram.

### Prognostic value of novel index HALPA

**Figure 5:**
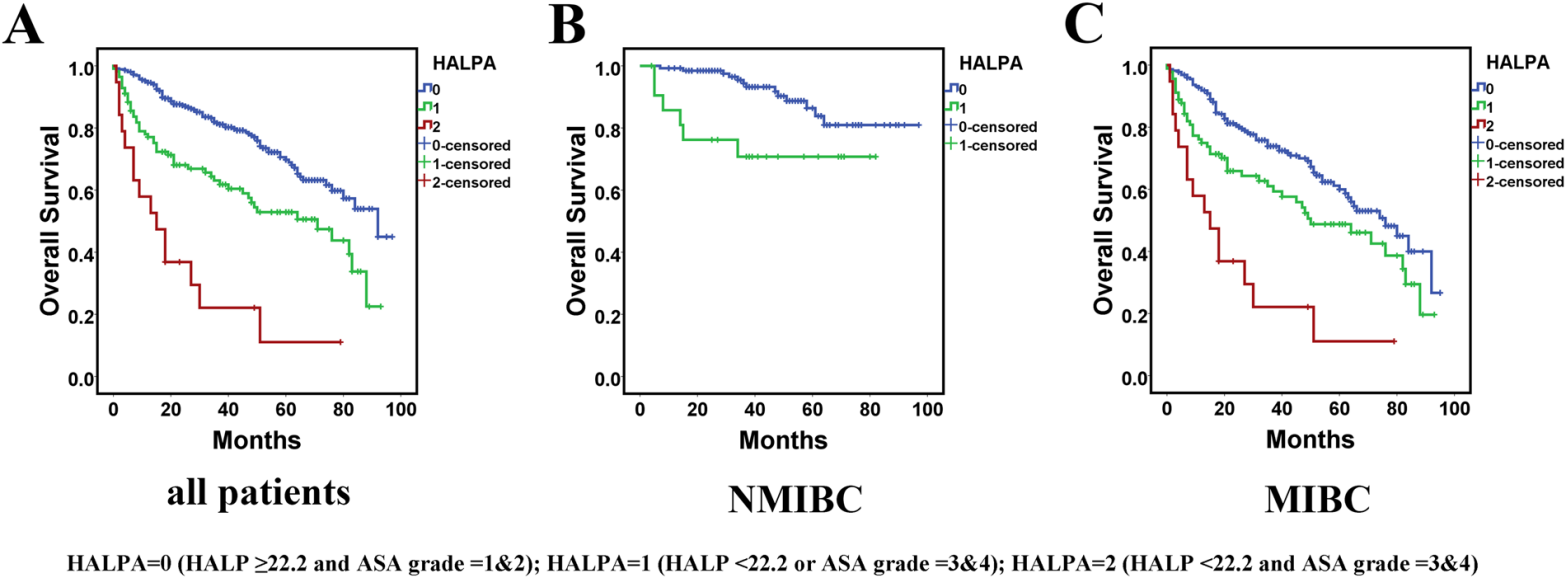
Kaplan-Meier curves for all patients (**A**), NMIBC patients (**B**), MIBC patients (C) by HALPA score.

In the multivariate analysis and nomograms, ASA was a significant factor in addition to TNM stage and HALP score. Therefore, we combined ASA grade and HALP score and created a new index, HALPA score. The score of HALPA was determined as follows: HALPA = 0 (HALP ≥ 22.2 and ASA grade = 1&2); HALPA = 1 (HALP < 22.2 or ASA grade = 3&4); and HALPA = 2 (HALP < 22.2 and ASA grade = 3&4). Therefore, a higher HALPA score indicated higher risk of decreased survival. Univariate and multivariable analyses were performed again with HALPA score. On log-rank test, HALPA score was a significant indicator of poor survival for all patients and NMIBC or MIBC patients (Fig. 5). Notably, no NMIBC patient had a HALPA score of 2, which also showed the ability of HALPA score to distinguish the tumor stage of bladder cancer patients. On multivariable analysis, high HALPA score remained an independent risk factor of decreased survival along with older age and TNM stage (HALPA score = 1, HR = 1.624, 95% CI:1.139–2.314, P = 0.007; HALPA score = 2, HR = 3.471, 95% CI: 1.861–6.472, P < 0.001) (Table 4).

**Table 4:** Univariate and multivariable analysis of factors associated with overall survival in bladder cancer patients who underwent radical cystectomy including HALPA.

Variable	Univariate analysis		Multivariate analysis	
	HR (95% CI)	*P* value	HR (95% CI)	*P* value
Gender (female vs. male)	0.794(0.528–1.193)	0.264		
Age (>65 vs. ≤65)	2.220(1.592–3.098)	**0.001**	1.849 (1.294–2.641)	**0.001**
Smoking history	1.014(0.725–1.417)	0.937		
Alcohol-drinking history	0.922(0.565–1.504)	0.743		
Hypertension	1.130(0.803–1.591)	0.480		
Histology type (TCC vs. no-TCC)	0.706(0.331)1.506	0.363		
G (3 vs. 2)	2.118(1.373–3.267)	**<0.001**		
T (MIBC vs. NMIBC)	4.301(2.665–6.942)	**<0.001**	2.831 (1.720–4.658)	**<0.001**
N (positive vs. negative)	2.448(1.716–3.491)	**<0.001**	2.135 (1.448–3.146)	**<0.001**
M (positive vs. negative)	10.509(3.842–28.746)	**<0.001**	3.807 (1.278–11.336)	**0.016**
Adjunctive chemotherapy	2.056(1.437–2.941)	**<0.001**		
ASA (3&4 vs. 1&2)	2.068(1.442–2.964)	**<0.001**		
Hypoalbuminemia	2.919(1.900–4.484)	**<0.001**		
Anemia	2.433(1.775–3.335)	**<0.001**		
PLR	1.963(1.421–2.712)	**<0.001**		
HALP	2.820(1.976–4.025)	**<0.001**		
HALPA		**<0.001**		**<0.001**
0	1		1	
1	2.084(1.477–2.941)	**<0.001**	1.624 (1.139–2.314)	**0.007**
2	6.300(3.628–10.940)	**<0.001**	3.471 (1.861–6.472)	**<0.001**

TCC, transitional cell carcinoma; MIBC, muscle-invasive bladder cancer; NMIBC, non-muscle invasive bladder cancer; ASA, American Society of Anesthesiologists; PLR: platelet to lymphocyte ratio; HR, hazard ratio; CI: confidence interval.

## Discussion

Besides traditional TNM stage and histologic classification, several haematological-based indices such as hemoglobin, albumin, and CRP levels, NLR, PLR and LMR were found associated with outcome with bladder cancer^7,10,12–14,17^. In the present study, we evaluated the prognostic value of a recently reported index, HALP, which combined hemoglobin and albumin levels and lymphocyte and platelet count, and found it as a good prognostic index for overall survival of patients with bladder cancer after radical cystectomy. By combining ASA grade and HALP score, we also created HALPA score and found it as an independent risk factor of decreased overall survival.

Cancer-related anemia is a common complication for cancer patients because of chromic blood loss, iron deficiency, vitamin deficiency and inflammation imbalance in terms of increased expression of interleukin and tumor necrosis factor. Jung *et al*.^7^ reported that 81 of 200 (40.5%) bladder cancer patients undergoing radical cystectomy had preoperative anemia and that anemia was significantly associated with disease recurrence and cancer-specific mortality and for MIBC patients, 53.3% patients were anemic, and older age, neoadjuvant chemotherapy, female sex, and low BMI were independent predictors of preoperative anemia. In this study, anemia was present in 27.9% patients (12.4% NMIBC, 37.5% MIBC, P < 0.001), which was lower than in previous reports. This difference may be due to use of different population. On univariate analysis, anemia was significantly associated with decreased overall survival (P < 0.001).

Hypoalbuminemia is another nutrition deficiency index. Lambert *et al*.^11^ reported that for bladder cancer patients after radical cystectomy, 16.5% had low-albumin level (<3.5 g/dL), and low albumin level was significantly associated with increased risk of mortality (HR = 1.76, P = 0.04). Another study found that 197 of 1471 (13.4%) bladder cancer patients had a low serum albumin level, and low albumin level was independently associated with reduced recurrence-free survival (HR 1.68, P = 0.006) and overall survival (HR 1.93, P < 0.001). Moreover, the authors also found low serum albumin associated with higher 90-day complication rate (42% vs. 34%, P = 0.03) and 90-day mortality (7.6% vs. 3.3%, P = 0.008)^17^. These findings document the adverse effects of nutritional deficiency on survival of bladder cancer after radical cystectomy and demonstrate the importance of nutritional interventions.

Tumor-promoting inflammation is one of the hallmarks of cancer^18^. For example, neutrophils and platelets could promote carcinogenesis, angiogenesis, invasion, or metastasis by secreting proinflammatory cytokines^19,20^. However, lymphocytes could inhibit tumor proliferation and migration via cytotoxicity^21^. Therefore, high PLR level, which reflects high platelet count and low lymphocyte count was found associated with overall survival in patients with bladder cancer undergoing radical cystectomy^14^.

The association of ASA score and long-term survival outcomes of bladder cancer patients after radical cystectomy is rarely reported. One study found that 740 of 1964 (64.8%) patients who underwent radical cystectomy had a high ASA score (3 or 4), and a high ASA grade remained independently associated with decreased overall survival (HR 1.45, 95% CI: 1.13–1.86, P = 0.003)^17^. In this study, a high ASA score was also significant independent risk factor for decreased overall survival and the nomogram including ASA grade and HALP score had better predictive accuracy than TNM stage. By combining ASA grade and HALP score, we created a novel index, HALPA score, and found it to have a specific distribution in patients at different stages and a significant independent risk factor with higher risk ratio than HALP score or ASA alone.

There are several limitations to this study. First, the retrospective nature has inherent limitations. Second, this was a single-center study and could not avoid the bias of population choice. Third, the use of cutoffs for continuous variables might weaken the reliability of the risk system. Finally, ASA is a subjective measure and this may limit the generalizability and overall utility. The prognostic ability and accuracy of HALP and HALPA score for bladder cancer patients warrants prospective multi-center validation.

## Conclusions

HALP may be a good prognostic index for overall survival for patients with bladder cancer after radical cystectomy. Combining ASA grade and HALP score in the HALPA score could distinguish tumor stage and was an independent risk factor for decreased overall survival. These indices could be used for risk stratification of individual bladder cancer patients and for choosing therapeutic strategy.

## Methods

### Data collection

This study was approved by the Institutional Review Board of Peking University First Hospital. Between 2006 and 2012, 516 bladder cancer consecutive patients underwent radical cystectomy in the Department of Urology, Peking University First Hospital. This research was carried out in accordance with the approved guidelines and informed consent was obtained from all patients. Hematological features including hemoglobin and albumin levels and blood cell counts were preoperative collected within 3 days before surgery. Other clinicopathological data included gender, age, smoking history, hypertension history, histology subtype, grade, TNM stage and American Society of Anesthesiologists (ASA) grade.

The follow-up of bladder cancer patients after radical cystectomy was according to the routine procedure in our institution. The period from the operation date until the time of death resulting from any cause was calculated as overall survival time. HALP was calculated as hemoglobin (g/L) × albumin (g/L) levels × lymphocyte count (/L)/platelet count (/L) and PLR as platelet count/lymphocyte count. Anemia was defined according to preoperative hemoglobin level (males <120 g/L and females <110 g/L) and hypoalbuminemia according to preoperative albumin level (<35 g/L).

### Statistical analysis

X-tile software v3.6.1 (Yale University) was used to determine the cut-off values of PLR and HALP^22^. SPSS v22.0 (SPSS Inc., Chicago, IL, USA) was used for all statistical analyses. Chi-square test was used to evaluate the association between clinicopathological data and HALP. Overall survival was estimated by the Kaplan-Meier method and the log-rank test was used for univariate analysis. Variables with significant differences on univariate analysis (P < 0.05) were then included in the multivariable survival analyses with a Cox proportional-hazards regression model, estimating hazard ratios (HRs) and 95% confidence intervals (CIs). Nomogram and production of calibration curves involved use of R v3.3.2 (R Foundation for Statistical Computing) with the rms package (Regression Modeling Strategies). A two-tail P < 0.05 was considered statistically significant.
